# The Validity and Between-Unit Variability of GNSS Units (STATSports Apex 10 and 18 Hz) for Measuring Distance and Peak Speed in Team Sports

**DOI:** 10.3389/fphys.2018.01288

**Published:** 2018-09-21

**Authors:** Marco Beato, Giuseppe Coratella, Adam Stiff, Antonio Dello Iacono

**Affiliations:** ^1^School of Science, Technology and Engineering, University of Suffolk, Ipswich, United Kingdom; ^2^Department of Biomedical Sciences for Health, University of Milan, Milan, Italy; ^3^Institute of Clinical Exercise and Health Science, School of Health and Life Sciences, University of the West of Scotland, Hamilton, United Kingdom

**Keywords:** training, soccer, team sports, velocity, GPS

## Abstract

The aims of this study were (i) to investigate the criterion validity (vs. gold standard measurements) of the 10 and 18 Hz STATSports Apex units for measuring distances and peak speed (Vpeak) outcomes and (ii) to investigate the between-unit variability. Twenty university students were enrolled in the study (age 21 ± 2 years, weight 72 ± 6 kg, and height 1.76 ± 0.05 m). The criterion validity was tested by comparing the distances recorded by the units with ground truth reference (400-m trial, 128.5-m circuit, and 20-m trial). Vpeak values were compared with those determined by a gold standard criterion device (Stalker ATS Radar Gun) during a linear 20-m sprint. The distance biases for the Apex 10 Hz in the 400-m trial, 128.5-m circuit, and 20-m trial were 1.05 ± 0.87%, 2.3 ± 1.1%, and 1.11 ± 0.99%, respectively, while for the Apex 18 Hz the biases were 1.17 ± 0.73%, 2.11 ± 1.06%, and 1.15 ± 1.23%, respectively. Vpeak measured by the Apex 10 and 18 Hz were 26.5 ± 2.3 km h^-1^ and 26.5 ± 2.6 km h^-1^, respectively, with the criterion method reporting 26.3 ± 2.4 km h^-1^, with a bias of 2.36 ± 1.67% and 2.02 ± 1.24%, respectively. This study is the first to validate and compare the STATSports Apex 10 and 18 Hz. Between-analysis (*t*-test) for total distance and Vpeak reported non-significant differences. Apex units reported a small error of around 1–2% compared to the criterion distances during 400-m, 128.5-m circuit, 20-m trials, and Vpeak. In conclusion, both units could be used with confidence to measure these variables during training and match play.

## Introduction

In team sport context, the common technologies utilized to quantify external load parameters are: global navigation satellite systems (GNSS), video tracking systems (VTS), and local positioning systems (LPS/radar) (Di Salvo et al., 2006; Buchheit et al., 2014a; Beato and Jamil, 2018). Previous investigations have employed such technologies to evaluate training and match load responses in team sport modalities like soccer, rugby, hurling, etc., (Coutts and Duffield, 2010; Jennings et al., 2010; Cummins et al., 2013; Vickery et al., 2014; Beato et al., 2017a; Young et al., 2018). The main applications of such technological solutions consist in the ability to collect and further analyze distance-driven and positional measures like: total distances covered (TD), peak speed (Vpeak), and high intensity running efforts during training sessions and matches (Varley et al., 2012; Cummins et al., 2013; Vickery et al., 2014). As commonly agreed among practitioners, GNSS devices are less time-consuming during training sessions and matches compared to VTS and LPS (Carling et al., 2008; Beato and Jamil, 2018). Sport scientists can use GNSS information to give real-time feedback, considering the limited amount of time for post-processing analysis and the lesser amount of operator work required; for these reasons, GNSS represent the most common technology for the evaluation of external training load variables in team sports (Cummins et al., 2013; Beato et al., 2016; Buchheit and Simpson, 2017). It is well known that the evaluation of external load parameters has a critical impact on the coaching staffs’ decisions, daily made about the application of long-term periodization strategies (Mohr et al., 2005; Carling et al., 2008; Thorpe et al., 2017; Hoppe et al., 2018). However, GNSS has a number of technological and practical limitations that could affect the practitioners’ decisions making processes (Scott et al., 2016).

GNSS devices present large variability in accuracy among different manufacturers’ models (Coutts and Duffield, 2010; Beato et al., 2016; Scott et al., 2016). Therefore, an independent and rigorous scientific validation should be necessary when new hardware and software versions are released on the market. Previous investigations report that validity and reliability reference of specific GNSS units cannot be extended to other models released by either the same or a different manufacturer (Akenhead et al., 2014). The scientific literature reports that the validation process of a GNSS unit takes into consideration measures of: (i) validity, which explain the difference between the values recorded by the unit and a criterion measure and (ii) reliability, which refers to the reproducibility of values of a test on repeat occasions (Cummins et al., 2013; Malcata and Hopkins, 2014; Beato et al., 2017b). Previous studies have reported that higher sampling rates (e.g., 10 Hz) offer several advantages in terms of validity and reliability measures when compared to less powerful devices (1 and 5 Hz) (Scott et al., 2016). Higher accuracy in TD covered during both linear activities (e.g., forward running) and sports-specific circuits, and Vpeak have been reported for 10 Hz devices compared to 1 and 5 Hz ones (Scott et al., 2016).

Greater accuracy in the data collected may help sport scientists through understanding the players’ performances over a given training session, as well as small variations worth of interest in the players’ physical loads and efforts load among sessions. This is particularly relevant at professional and elite levels, where small differences can have a meaningful impact on performance outcomes throughout the season. By increasing the sampling frequency of the GNSS devices have resulted in several improvements in terms of data quality, however, some limitations continue to exist. Previous studies, employing 10 Hz GNSS devices, have shown low validity and reliability when tested in sport-specific circuits, as well as during high intensity short shuttle runs and change-of-direction maneuvers (Jennings et al., 2010; Johnston et al., 2014; Beato et al., 2016; Scott et al., 2016). Nevertheless, GNSS technology is under continuous development, and it is reasonable to assume that some recently released models could offer further advantages in accuracy compared to previous ones. This claim should be supported by a scientific validation study.

The STATSports Apex unit is an athlete-tracking system released in August 2017, and it is widely used in professional clubs (e.g., in the Premier League, Serie A, etc.). A previous validation of the STATSports Viper GNSS unit was performed in 2016, and it reported a distance bias of 2.5% during 20-m running activity and a small to moderate bias in speed (3–9%) during 5–20-m short shuttle runs (Beato et al., 2016). However, these values cannot be extended from the Viper GNSS unit to the Apex GNSS unit, sampling at 10 and 18 Hz. The STATSports Apex is available in two different device specifications. A 10 Hz multi-GNSS augmented unit is capable of acquiring and tracking multiple satellite systems [e.g., global positioning system (GPS), GLONASS, Galileo, and BeiDou] concurrently, and thus providing a more accurate positional information. Previous research has shown that the number of satellites connected to a tracking device plays a key role in GNSS accuracy, since there is a moderate negative correlation between the TD error and the number of visualized satellites interacting with the unit (Scott et al., 2016). The second model is the Apex 18 Hz unit, which can access GPS satellite system frequency bands but does not support space-based augmentation (it is based only on GPS); however, the sampling frequency has been increased (from 10 to 18 Hz) in comparison to that of the previous model. Sampling frequency has also been reported to be closely associated with data accuracy (Scott et al., 2016). Both technological improvements should offer advantages in term of unit validity. Moreover, in the previous version of the STATSports units (Viper 10 Hz), relevant information such as the number of satellites visualized and horizontal dilution of precision data was missing, while is currently provided in the latest released Apex units.

To date, no validation and comparison studies of the Apex 10 and 18 Hz exist; therefore, it would be acknowledgeable to report accuracy data of such units and existing differences between the models, which could offer important practical applications for practitioners working in sport contexts. This need is further required when considering that GNSS-derived data is utilized to manage players’ training loads, recovery strategies, workload implementations, and subsequent training periodization (Buchheit et al., 2014a; Brown et al., 2016; Christopher et al., 2016). The validation process is a crucial step for the application of GNSS (Apex 10 and 18 Hz) in team sports, while information concerning the accuracy of such devices could offer additional benefits to training-load analysis in research studies (Malone et al., 2015; Beato et al., 2017b). It is also fundamental to understand the validity of STATSports Apex units, to better recognize the variability in the metrics used during training sessions and for making comparisons among the players in order to optimize the training process and the players’ workload periodization (Thorpe et al., 2015, 2017). Such interpretations and decisions can be made only when the validity of a technology being utilized is established. Therefore, the aims of this study were to assess the validity of STATSports Apex (10 and 18 Hz) units, as well as to investigate the between-unit variability by evaluating distances and Vpeak during sports-specific activities.

## Materials and Methods

### Participants and Research Design

Twenty physical active male and female university students were enrolled (age 21 ± 2 years, weight 72 ± 6 kg, and height 1.76 ± 0.05 m) in this descriptive study (data recorded in 2018). The experimental protocol was in accordance with the Declaration of Helsinki for study on human subjects. The Institutional Ethics Board of the University of Suffolk (Ipswich, United Kingdom) approved the experimental protocol. A written informed consent was obtained from the participants of this study.

### Experimental Protocol and Data Analysis

**FIGURE 1:**
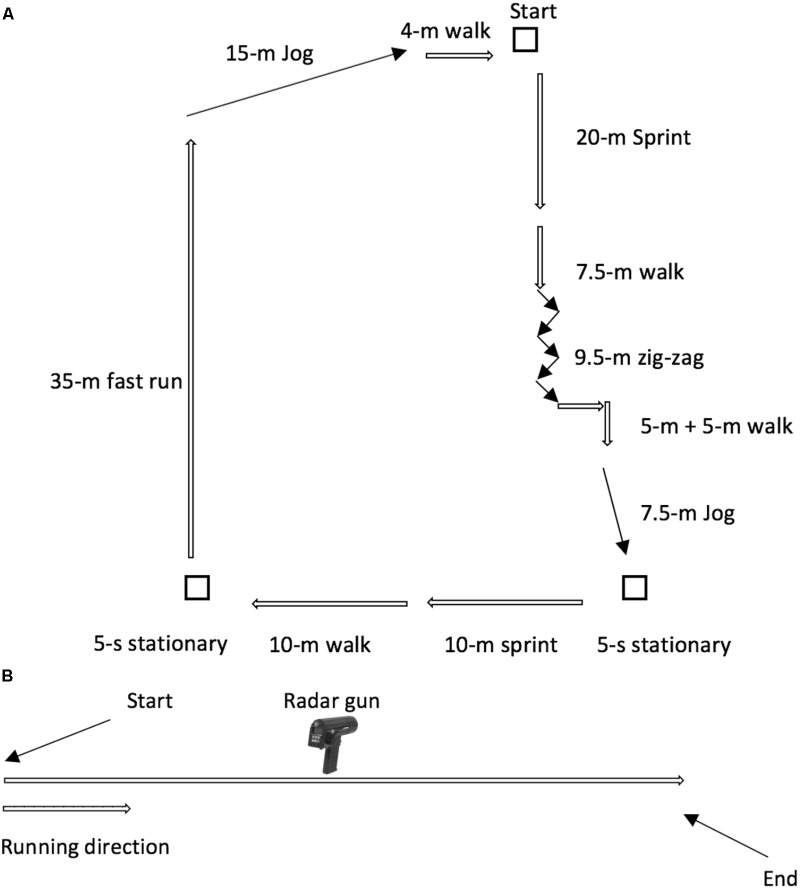
**(A)** Specific team sports circuit of 128.5 m. **(B)** Sprint 20-m. Marco Beato, Gavin Devereux, and Adam Stiff, Validity and Reliability of Global Positioning System Units (STATSports Viper) for Measuring Distance and Peak Speed in Sports, Journal of Strength and Conditioning Research, volume and issue number not available as at the moment of reuse the article was still Publish Ahead of Print (PAP), August 2018, Wolters Kluwer ll Rights Reserved https://journals.lww.com/nsca-jscr/Abstract/publishahead/Validity_and_Reliability_of_Global_Positioning.95218.aspx.

GNSS (STATSports Apex, Northern Ireland) data were collected outdoor on an athletic track, in absence of high and large buildings in the surrounding area to enhance satellites’ reception (Williams and Morgan, 2009). Both Apex 10 and 18 Hz were connected with a number of satellites of 20, ranged between 18 and 21, while the horizontal dilution of precision during the trials was 0.4 ± 0 for both Apex models. GNSS accuracy for evaluating distance was tested against the criterion distance of a 400-m athletic track, a specific team sports circuit of 128.5-m that replicated the movement demands of team sports (circuit performed on synthetic surface, **Figure 1A**), and a 20-m trial (linear running) (Hoppe et al., 2018). The participants were instructed to remain in a standing position for 30 s, after beginning the experimental trials. All subjects returned exactly to the starting point and then waited for another 30 s in a standing position. The start time for each trial was determined by the increase above zero on the velocity trace. GNSS data validity was evaluated by comparing the instantaneous values of speed (Vpeak) collected by these devices and with those determined by a radar gun (Stalker ATS 2, 34.7 GHz, United States) during a 20-m sprint (**Figure 1B**). ATS II radar uses high frequency radio waves and measures changes of speed of a moving object (Doppler radar). Radar gun and laser devices are considered to be gold standard instruments for evaluating Vpeak (Varley et al., 2012; Cummins et al., 2013; Scott et al., 2016). The radar device was set to measure forward sprinting velocity and was operated remotely via laptop connection to negate the possibility of variability introduced due direct manual operation (Cross et al., 2015). The speed data were analyzed (after an instantaneous filtering; Dig Medium, moving average five points) using the Stalker ATS Version 5.0.3.0 software (Beato et al., 2018). Stalker ATS validity and reliability were previously reported (Haugen and Buchheit, 2016).

Participants completed a 400-m trial at a self-selected speed (jogging pace), a 128.5-m trial, a 20-m trial (jogging pace), and a 20-m sprint at maximum speed. Each participant was verbally instructed before each trial to perform the correct procedure. Participants performed a familiarization trial (week 1) before the beginning of the experimental trial. A 400-m trial, 128.5-m circuit, 20-m trial, and 20-m sprint were performed by the participants of this study (validity evaluation, week 2). The experimental session was performed during a sunny day without rain or clouds.

**FIGURE 2:**
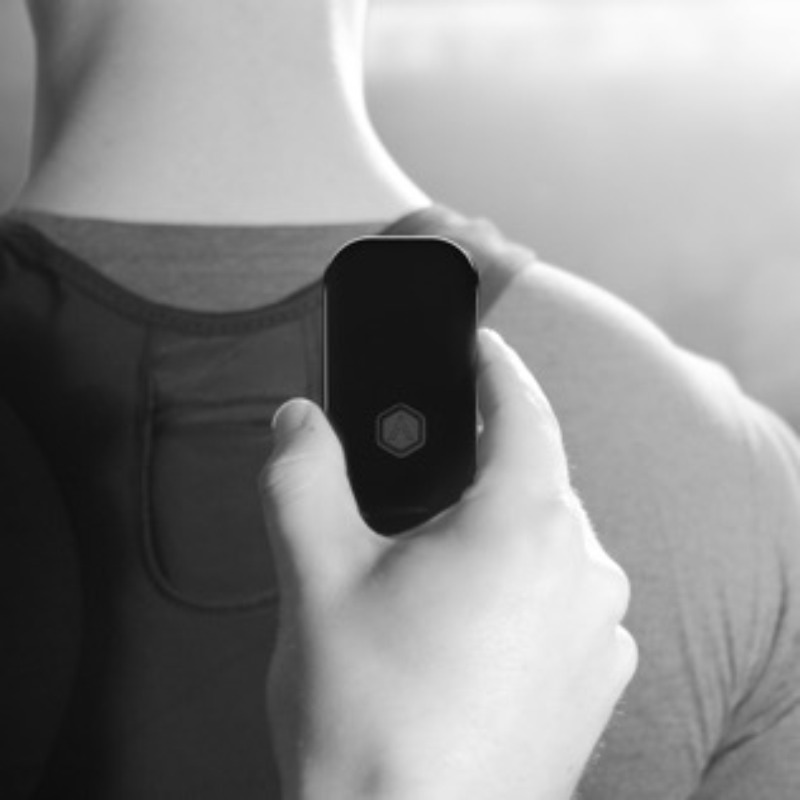
STATS ports Apex unit and harness.

The Apex units (10 and 18 Hz) were turned on about 10–15 min before the beginning of the test, while the subjects were familiarized with the equipment as well as the protocol procedures. Apex units present the following characteristics: dimension 30 mm (wide) × 80 mm (high), weight 48 g, 100 Hz gyroscope, 100 Hz tri-axial accelerometer, and 10 Hz magnetometer. Prior to the experiments, both Apex unit models (Apex 10 and 18 Hz, STATSports, Northern Ireland) were placed on the back of the participant, midway between the scapulas (**Figure 2**). Apex 10 Hz is a multi-GNSS augmented unit, which is capable of acquiring and tracking multiple satellite systems (e.g., GPS, GLONASS, Galileo, BeiDou) concurrently to provide the best possible positional information. In contrast, Apex 18 Hz units have a higher sampling frequency than Apex 10 Hz, but its acquisition system is based only on GPS. GNSS data (speed and distance) recorded by the units were downloaded and further analyzed by the STATSports Apex Software (Apex 10 Hz version 2.0.2.4 and Apex 18 Hz version 5.0, respectively).

### Statistical Analyses

A total of 80 trials were analyses in this study. Data are presented as means ± SD. A Shapiro–Wilk test was performed for the evaluation of normality (assumption) of the statistical distribution. Validity was assessed by calculating the bias (%) between the known distance and the unit (absolute error). Bias was interpreted as poor (>10%), moderate (5–10%), or good (<5%) (Hopkins et al., 2009). A paired *t*-test was used to compare the differences in TD covered during the 400-m and 128.5-m circuit, 20-m trial and Vpeak recorded between the Apex models. Statistical significance was set at p < 0.05. Differences between the units and a criterion measure were reported as a mean change with confidence intervals (CI 90%) (Hopkins, 2000). Effect size was interpreted by Cohen as trivial <0.2, small 0.2–0.6, moderate 0.6–1.2, large 1.2–2.0, and very large >2.0 (Cohen et al., 1990). Intraclass correlation (ICC) was used to compare criterion and Apex Vpeak. An interpretation system from trivial (<0.1), small (0.1–0.3), moderate (0.3–05), large (0.5–07), very large (0.7–0.9), nearly perfect (0.9), to perfect (1.0) scores was used (Hopkins et al., 2009). The between-unit variability between Apex 10 and 18 Hz was assessed using the typical error of measurement and expressed as percentage of coefficient of variation (CV). Statistical analysis was performed using SPSS (Statistics 20.0) for Mac OS Sierra (version 10.12.5).

## Results

The Shapiro–Wilk test confirmed the assumption of normality of the statistical distribution. Apex 10 Hz GNSS distance covered in the 400-m trial, 128.5-m circuit, and 20-m trial was 398.7 ± 7.6 m, 131.4 ± 1.4 m, 20.07 ± 0.29 m, respectively, with an absolute error of 4.19 ± 3.48 m, 2.85 ± 1.4 m, and 0.22 ± 0.20 m, respectively. The bias in each trial was 1.05 ± 0.87%, 2.3 ± 1.1%, and 1.11 ± 0.99%, respectively. Vpeak measured by the Apex 10 Hz was 26.5 ± 2.3 km h^-1^ and criterion was 26.3 ± 2.4 km h^-1^. Mean difference (90% CI) was 0.17 (-0.18; 0.53), *t* = 1.012, *p* = 0.32, ES (90% CI) = 0.08 (-0.05; 0.22), trivial. The absolute error of the Apex 10 Hz was 0.62 ± 0.45 km h^-1^ and the bias was 2.36 ± 1.67% (good). ICC between Apex 10 Hz and radar gun Vpeak was nearly perfect [*p* < 0.001, ICC (90% CI) = 0.96 (0.92; 0.98)].

Apex 18 Hz distance covered in the 400-m trial, 128.5-m circuit, and 20-m trial was 396.8 ± 11.6 m, 131.2 ± 1.3 m, and 20.19 ± 0.28 m, respectively, with an absolute error of 4.7 ± 2.9 m, 2.7 ± 1.4 m, and 0.23 ± 0.25 m, respectively. The bias in each trial was 1.17 ± 0.73%, 2.11 ± 1.06%, and 1.15 ± 1.23%, respectively. Vpeak measured by the Apex 18 Hz was 26.5 ± 2.6 km h^-1^ and criterion was 26.3 ± 2.4 km h^-1^. Mean difference (90% CI) was 0.24 (-0.15; 0.50), *t* = 1.973, *p* = 0.064, ES (90% CI) = 0.08 (0.01; 0.15), trivial. The absolute error of the Apex 18 Hz was 0.52 ± 0.30 km h^-1^ and the bias was 2.02 ± 1.24% (good). ICC between Apex 18 Hz and radar gun Vpeak was nearly perfect [*p* < 0.001, ICC (90% CI) = 0.98 (0.96; 0.99)].

Between-analysis (Apex 10 Hz vs. Apex 18 Hz) did not find any meaningful differences in the 400-m trial (mean change, CI 90%) 1.78 (-2.9; 6.5), *t* = 0.653, *p* = 0.522; in the 128.5-m circuit of 0.15 (-0.65; 0.94), *t* = 0.319, *p* = 0.753; in the 20-m trial of -0.12 (-0.27; 0.02), *t* = 1.45, *p* = 0.163; or in the 20 m sprint Vpeak of -0.04 (-0.38; 0.23), *t* = 0.403, *p* = 0.691, ICC (CI 90%) = 0.96 (0.91; 0.98).

## Discussion

The aims of this study were to validate and to compare the STATSports units (Apex 10 and 18 Hz) when measuring distance and Vpeak outcomes during sports-specific activities. The main findings of this research were that the STATSports Apex (10 and 18 Hz) reported a small bias (<5%) for distance measures (400-m trial, 128.5-m circuit, and 20-m trial) and Vpeak (20 m sprint), thus supporting the validity of both Apex models. Trivial (ES) differences were found between the Apex 10 and 18 Hz in the parameters analyzed (distance covered in 400-m trial, 128.5-m circuit, and 20-m trial, as well as Vpeak in the 20-m trial) (**Table 1**).

**Table 1:** Between-analysis (Apex 10 Hz vs. Apex 18 Hz) during 400-m trial, 128.5-m circuit, 20-m trial and 20-m sprint (20 players). Data are presented in mean ± SD.

Variables	Apex 10 Hz	Apex 18 Hz	Mean difference (CI 90%)	CV (CI 90%)	P-level	ES (CI 90%) Qualitative
400-m Distance (m)	398.7 ± 7.6	396.8 ± 11.6	1.78 (-2.9; 6.5)	2.0 (1.5; 2.8)	0.522	0.18 (-0.3;0.66) trivial
128.5-m Distance (m)	131.4 ± 1.4	131.2 ± 1.3	0.15 (-0.65; 0.94)	1.1 (0.9; 1.5)	0.753	0.11 (-0.49; 0.71) trivial
20-m Distance (m)	20.07 ± 0.29	20.19 ± 0.28	-0.12 (-0.27; 0.02)	1.3 (1.0; 1.8)	0.163	0.42 (-0.08; 0.92) small
Vpeak 20-m (km h^-1^)	26.51 ± 2.3	26.55 ± 2.6	-0.04 (-0.38; 0.23)	2.3 (1.8; 3.3)	0.691	0.03 (-0.09; 0.16) trivial

*Vpeak, peak speed; SD, standard deviation; CI, confidence intervals; CV, coefficient of variation; ES, effect size*.

GNSS is a technology commonly used to evaluate external training load (e.g., TD, Vpeak, accelerations, sprints, etc.) in team sports (Buchheit et al., 2014a; Rampinini et al., 2015; Akenhead and Nassis, 2016; Beato and Jamil, 2018; Hoppe et al., 2018). Previous studies reported that GNSS devices have low accuracy during short shuttle runs, change of directions, and high-intensity activities (Buchheit et al., 2014a,b; Stevens et al., 2014). The current study analyzed the validity of the STATSports Apex (10 and 18 Hz) resulting in contrasting findings (Cummins et al., 2013). The parameters analyzed in this study were TD and Vpeak in three different activities. However, small biases were reported for the parameters analyzed. Several studies have highlighted that the sampling rate is a crucial factor associated with validity and reliability (Coutts and Duffield, 2010; Hoppe et al., 2018). Higher sampling frequency devices (10–15 Hz) are more accurate and reliable than 1–5 Hz units (Scott et al., 2016). Based on such evidence, it seems logical to assume that by increasing the sampling rate it resulted into an improvement of the validity of the new Apex 18 Hz unit, especially during the high-intensity activities (e.g., Vpeak) that athletes perform during training sessions and matches (Beato et al., 2016; Christopher et al., 2016; Hoppe et al., 2018). Both Apex models employed in the current research (10 and 18 Hz) have a higher sampling rate than many other devices previously analyzed (1–5 Hz) (Cummins et al., 2013).

Apex reported lower or equal bias than previous wearable devices analyzed, such as 9.6–32.4% in TD (20 m sprint) MinimaxX team (1 Hz), 1.7–6.7% in TD (30 m sprint) MinimaxX v4.0 (10 Hz), and 2.9–7.7% in TD (30–40 m print) SPI-Pro (5 Hz) (Scott et al., 2016). A recent study reported the validity of MinimaxX (S4 10 Hz) presenting a bias (%) of 3.3, 2.1, and 6.8% in 10 m jogging, 129.6 m circuit, and 30 m sprint (Hoppe et al., 2018). The same study reported the validity of GPXE PRO (18 Hz) that presented a bias (%) of 2.2, 1.6, and 6.7% in 10-m walking, 129.6-m circuit, and 30-m sprint, respectively. Previous STATSports Viper units presented a small bias (2.53%) during a 20-m trial (Beato et al., 2016). This data agreed with the previous knowledge that GNSS has lower accuracy during high-intensity short distance activities than in longer-distance trials (Scott et al., 2016).

In the current study, both Apex 10 and 18 Hz units resulted in small bias in distance measures during the 400-m, 128.5-m, and 20-m trials, as well as in Vpeak during the 20-m sprint. These scores are smaller than the bias reported for TD and Vpeak of previous Viper models. A previous study, employing the STATSports Viper (10 Hz) units, found a bias ranging from of 8.7 to 3.4% (moderate to small) in speed outcomes during 5–20-m running activity associated to this model (Beato et al., 2016). Several factors could explain these improvements, such as a higher sampling rate (Apex 18 Hz) and higher number of satellites available and mitigated ionosphere errors (Apex 10 Hz). In the current study, the same number of satellites (average 20, ranged 18–21) and horizontal dilution of precision were found during the trials (0.4 ± 0) for both Apex models. As reported above, the STATSports Apex 10 Hz device utilizes a multi-band GNSS receiver in combination with corrected signal information from space-based augmentation systems to achieve enhanced data quality. These technological improvements could explain the lower bias reported in the current research compared to the previous STATSports Viper units (Beato et al., 2016). However, such improvement in accuracy could be related not only to technological improvements, but also to differences in the protocols utilized. When Viper units have been analyzed, participants performed short shuttle runs involving change of directions at different speeds. It is well known that a 180° change of direction can highly affect accuracy, therefore a comparison between the studies should be done with caution. Moreover, these differences could be induced by the individual variability of the performance of the participants recruited in the two studies, since the abilities to replicate the changes of direction could not be consistent across the studies. Furthermore, the criterion speed was measured by using different methodologies between the two studies, such as video analysis and radar gun (Beato et al., 2016). It is largely accepted that video analysis is not a gold standard method for evaluating Vpeak or average speed during linear movements, as radar technology is considered to be (Scott et al., 2016). The differences among the units and the criterion speeds reported in this study were trivial, therefore sports scientists and coaches could use these models to evaluate athletes’ speed interchangeably. In field contexts, it is very rare to have a radar gun or laser technology available to evaluate players’ Vpeak. The results reported in this study showed that both Apex units (10 and 18 Hz) could be utilized to assess sprint performance in team sports (Roe et al., 2017; Hoppe et al., 2018). Our current results agreed with a recent publication drawing the same conclusion and reporting that 10 Hz GNSS provide valid measures of 40-m Vmax assessment when compared with a radar gun (Roe et al., 2017). Sports scientists and coaches could integrate Vpeak tests in their fitness battery, as well as evaluate Vpeak during training sessions or matches, using GNSS technology.

An innovative finding of the current study is the comparison between Apex 10 and 18 Hz on TD and Vpeak. The between-unit analysis (Apex 10 Hz vs. Apex 18 Hz) found trivial to small differences in the 400-m trial (ES = 0.18), 128.5-m circuit (ES = 0.11), 20-m trial (ES = 0.42), and in 20-m sprint Vpeak (ES = 0.03). Following these results, it may be concluded that these two Apex models do not present meaningful differences in the parameters evaluated, therefore sport scientist and coaches could use both models interchangeably in their practice (even if consistency in the model utilization should be recommended). This is the first study to evaluate and compare such units, thus it is not possible to make any comparisons with other research work published. A future study could replicate the current study, analyzing Apex units.

This study presents three main limitations: firstly, we evaluated sports-specific movements by linear running activity and circuits involving human participants. It is well known that this approach (largely used in the literature) might present errors, and therefore the results reported in the current study should be considered carefully. For instance, during the 400-m trial and the 128.5-m circuit, the variability due to the human movement inconsistency could have affected the findings. Sports scientists and coaches should consider that research studies involving human subjects might show some inconsistency between the designs. The application of mechanical devices collecting at higher frequency than the human movement could be used to test distance and speed. Secondly, the Vicon motion analysis system has been recently proposed as the new gold standard technology for evaluating sports-specific movements (Scott et al., 2016). Such technology could offer some advantages compared to the laser and radar devices that are employed to evaluate Vpeak during linear activities. It is well known that laser and radar technology cannot be used during sports- movements, therefore Vicon (or similar devices) could offer additional information about GNSS validity and reliability (Scott et al., 2016). Another limitation may be associated with the research conditions. Data reported in this study were obtained in optimal conditions, thus they cannot be extended to every environmental condition. For example, many soccer and rugby teams use such GNSS-based athlete monitoring devices during official competitions, however, previous studies found that nearby high buildings could affect the validity and reliability of the data recorded in these environments. Coaches and sport scientists should interpret GNSS data with caution when it has been recorded in suboptimal conditions (e.g., a stadium) (Witte and Wilson, 2004; Scott et al., 2016). Future studies could replicate the current study inside a stadium and therefore analyze Apex validity in such conditions.

### Practical Applications

The evaluation of GNSS Apex 10 and 18 Hz validity is a critical step for its application in team sports and for research purposes. Apex units are largely utilized in team sports (e.g., rugby, hurling, and soccer, please see STATSports website). This study provides innovative findings and offers important implications for sports scientists and researchers involved with such technologies for practical and research purposes. Apex units (10 and 18 Hz) showed good levels of accuracy (bias < 5%) in sport specific metrics. Practitioners can be mindful that the units analyzed in this study can be used to evaluate distance covered during linear running and sports-specific activity. Moreover, Apex units can be used to evaluate Vpeak in sports, since non-significant and trivial differences were found compared to criterion Vpeak (radar gun). External load interpretation and the associated decision making processes can only be made when the validity of GNSS technology is well known. Coaches and sport scientists can use the metrics derived from STATSports Apex units, which have been analyzed in this study, to quantify players’ workload during training sessions and to optimize the overall training periodization.

## Author Contributions

MB was the main investigator. GC was involved in the data analysis and statistics. AS had a critical role for data recording. AI was the supervisor of the project.

## Conflict of Interest Statement

AI was employed at the time of the study by Maccabi Tel Aviv FC. The remaining authors declare that the research was conducted in the absence of any commercial or financial relationships that could be construed as a potential conflict of interest. The handling Editor declared a past co-authorship with one of the authors AI.
